# Role of a Fur homolog in iron metabolism in *Nitrosomonas europaea*

**DOI:** 10.1186/1471-2180-11-37

**Published:** 2011-02-21

**Authors:** Neeraja Vajrala, Luis A Sayavedra-Soto, Peter J Bottomley, Daniel J Arp

**Affiliations:** 1 Department of Botany and Plant Pathology, 2082 Cordley, Oregon State University, Corvallis OR 97331, USA; 2 Department of Microbiology, 220 Nash, Oregon State University, Corvallis OR 97331, USA

## Abstract

**Background:**

In response to environmental iron concentrations, many bacteria coordinately regulate transcription of genes involved in iron acquisition via the ferric uptake regulation (Fur) system. The genome of *Nitrosomonas europaea*, an ammonia-oxidizing bacterium, carries three genes (NE0616, NE0730 and NE1722) encoding proteins belonging to Fur family.

**Results:**

Of the three *N. europaea fur *homologs, only the Fur homolog encoded by gene NE0616 complemented the *Escherichia coli *H1780 *fur *mutant. A *N. europaea fur:kanP *mutant strain was created by insertion of kanamycin-resistance cassette in the promoter region of NE0616 *fur *homolog. The total cellular iron contents of the *fur:kanP *mutant strain increased by 1.5-fold compared to wild type when grown in Fe-replete media. Relative to the wild type, the *fur:kanP *mutant exhibited increased sensitivity to iron at or above 500 μM concentrations. Unlike the wild type, the *fur:kanP *mutant was capable of utilizing iron-bound ferrioxamine without any lag phase and showed over expression of several outer membrane TonB-dependent receptor proteins irrespective of Fe availability.

**Conclusions:**

Our studies have clearly indicated a role in Fe regulation by the Fur protein encoded by *N. europaea *NE0616 gene. Additional studies are required to fully delineate role of this *fur *homolog.

## Background

The molecular basis for the coordinated regulation of iron acquisition systems by iron was first described for *Escherichia coli *[1]. Several bacteria are now known to regulate their iron acquisition systems via Fur (ferric uptake regulator) [2-5]. Fur is a sequence-specific DNA-binding protein that acts mainly as a negative regulator of transcription *in vivo *by complexing with ferrous (Fe^2+^) ion to repress the expression of iron-regulated genes [6]. Fur also activates the expression of many genes by either indirect or direct mechanisms [7]. Mutations in the *fur *gene resulted in constitutive expression of siderophores and outer membrane Fe^3+^-siderophore receptors potentially required for iron uptake [8].

*Nitrosomonas europaea *is an aerobic chemolithoautotroph that uses NH_3 _and CO_2 _for growth [9]. Mechanisms for iron transport are essential to this bacterium for maintaining the many cytochromes and other heme-binding proteins involved in ammonia metabolism [10,11]. The genome of *N. europaea *has 4% of its genes dedicated for iron acquisition, but no evidence for siderophore production was found [9]. *N. europaea's *inability to produce siderophores in Fe-replete or Fe-limited media was further confirmed by universal Chrome Azurol S assay [12]. *N. europaea *responds to iron limitation by elevating production of Fe^3+^-siderophore receptors normally repressed under iron-replete conditions [13,14]. Several *N. europaea *iron-repressible genes contain sequences similar to the *E. coli *Fur box (unpublished data) in their promoter regions; hence it is likely that a Fur-like repressor regulates iron uptake genes in *N. europaea *as well. Indeed, sequence annotation of *N. europaea *genome revealed three genes encoding *fur *homologs (NE0616, NE0730, NE1722) that contain characteristic Fur domains [9].

Multiple *fur *homologs have been described for several bacteria. Different species have a variable number of genes bearing the Fur domain. For example, *E. coli *[15] has two, *Bacillus subtilis *[16], *Mycobacterium smegmatis *have three, *Staphylococcus aureus *and some species of *Brucella *have four and *Thermoanaerobacter tengcongensis *has five *fur *homologs [17]. The apparent redundancy in *fur *homologs has been clarified by a considerable amount of experimental data obtained from genetic and biochemical analysis in bacteria such as *E. coli *and *B. subtilis *[15,16,18-20]. The experimental data suggests that the Fur protein family has several subclasses with different functions [19]. The major Fe-sensing Fur subclass is mainly involved in the control of iron homeostasis [21]. A second subclass controls the expression of genes involved in the response of bacteria to oxidative stress (i.e. PerR), but it does not appear to be involved in the cellular response to iron [16]. A third subclass called Zur (zinc uptake regulator) controls the uptake of zinc in *E. c*oli [15,20] and *B. subtilis *[18].

The Fe-sensing Fur protein has been extensively studied and is shown to act as a global regulator in response to environmental iron concentration due to its involvement in the regulation of activities as varied as the acid tolerance response, the oxidative stress response, metabolic pathways, and virulence factors [6]. In this study, we aimed to characterize the regulatory role of a *fur *homolog from *N. europaea*. Using genetic complementation studies, we demonstrated that one *fur *homolog (NE0616) out of three in *N. europaea *encoded a functional Fur protein. Here we report the construction of the *N. europaea fur *promoter knockout mutant (*fur:kanP*) strain, its effect on the expression of Fe-regulated proteins and the physiology of *N. europaea*.

## Results

### Sequence analysis of *N. europaea fur *homologs

**Figure 1:**
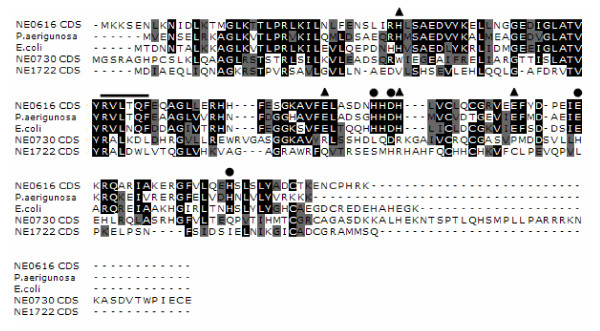
**Alignment of *N. europaea *Fur homolog coding sequences with *E. coli *and *P. aeruginosa *Fur proteins using ClustalW **[31]. Identical residues are shaded black, with similar residues shaded grey. Metal binding site 1 residues are indicated with circles, and site 2 residues are indicated with triangles, as identified from the crystal structure of *P. aeruginosa *Fur. Residues indicated by straight line highlight a motif thought to be involved in DNA binding.

**Figure 2:**
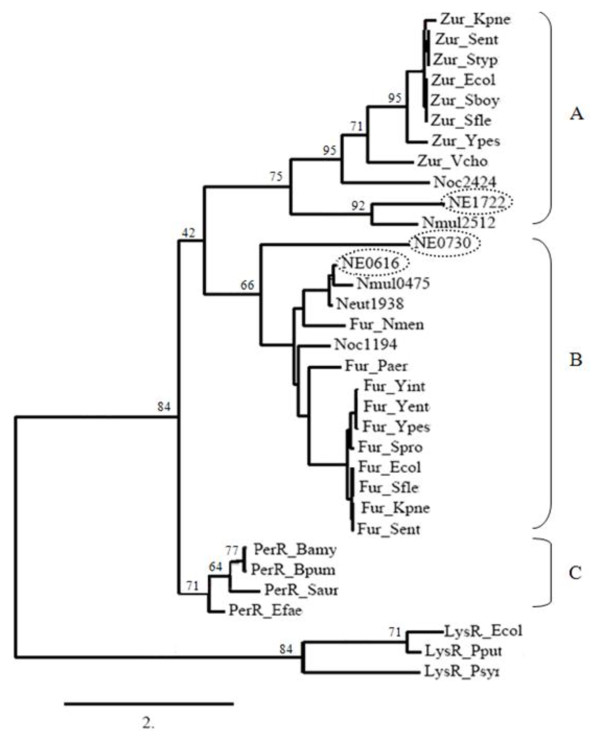
**Maximum-Likelihood tree of the Fur homologs. Phylogenetic tree of Fur encoding sequences generated by Phyml analysis**. The numbers beside nodes are the percentages of bootstrap values calculated for 200 replicates: The three groups - A, B and C - mentioned in the text are indicated on the right side of the tree. Bamy, *Bacillus amyloliquefaciens*; Bpum, *Bacillus pumilus*; Ecol, *Escherichia coli*; Efae, *Enterococcus faecalis*; Kpne, *Klebsiella pneumoniae*; Nmen, *Neisseria meningitidis*; Paer, *Pseudomonas aeruginosa*; Pput, *Pseudomonas putida*; Psyr, *Pseudomonas syringae*; Saur, *Staphylococcus aureus*; Sboy, *Shigella boydii*; Sent, *Salmonella enterica*; Sfle, *Shigella flexneri*; Spro, *Serratia proteamaculans***;**Styp, *Salmonella typhimurium*; Vcho, *Vibrio cholerae*; Yent, *Yersinia enterocolitica*; Yint, *Yersinia intermedia*; Ypes, *Yersinia pestis*; Ypse, *Yersinia pseudotuberculosis*; NE, *Nitrosomonas europaea*; Neut, *Nitrosomonas eutropha*; Nmul, *Nitrosospira multiformis*; Noc, *Nitrosococcus oceanii*.

The three *N. europaea *Fur-like repressors encoded by NE0616, NE0730, NE1722 are only distantly related to each other with 25% to 35% amino acid identity. The Fur homolog encoded by NE0616 is most similar (~84% similar to *E. coli *Fur protein) in sequence to various Gram-negative Fe-sensing Fur proteins. The publication of the crystal structure of the *Pseudomonas aeruginosa *Fur protein provided considerable insight into its 2 metal binding sites. Binding Site 1 represents the putative iron binding regulatory site and is coordinated by amino acids H86, D88, E107, and H124 and Site 2 is coordinated by H32, E80, H89 and E100 [19]. All these residues are conserved only in the *N. europaea *NE0616 Fur homolog but not in Fur homologs encoded by NE0730 and NE1722 (Figure 1). Phylogenetic analysis of Fur homolog coding sequences from *N. europaea *with Fur proteins from other bacteria placed NE0616 in the group B comprised of Fe-sensing Fur proteins, NE1722 in the group A comprised of Zn-sensing Zur proteins. Surprisingly, NE0730 Fur homolog was also placed in group B. No Fur homologs of *N. europaea *grouped with peroxide sensing PerR proteins i.e., in group C (Figure 2).

Based on well-studied model systems, expression of the *fur *gene itself is iron regulated and there is strong evidence that this is through a mechanism of autoregulation [34,35]. Fur recognizes and binds specifically to a DNA sequence, known as the Fur box, that is typically located in proximity to the -10 and/or -35 promoter elements of target genes [6]. Analysis of several Fur-binding sites allowed the early definition of a 19-bp inverted repeat consensus Fur box in *E. coli *[6]. Since then, canonical Fur boxes have been described in several bacteria such as *P. aeruginosa *[36], *Neisseria gonorrhoeae *[37] and *Vibrio cholerae *[38]. The canonical Fur box identified by *B. subtilis *Fur revealed a different conserved 15-bp (7-1-7) inverted repeat present twice within this 19-bp consensus sequence [39]. We have used an *in silico *approach, fed with experimentally confirmed *N. europaea *Fur boxes (unpublished data), to identify candidate Fur-binding sites in promoter regions of all 3 *N. europaea fur *homologs. A potential Fur box (5'-TAATAATACGTATCTTTAT-3') in the promoter region of NE0616 gene, -121 bp upstream of the proposed initiation of translation of the *fur *gene was found. We were unable to find potential Fur boxes in the promoter region of the other *N. europaea fur *homologs, NE0730 and NE1722.

### Complementation of an *E. coli fur *mutant by *N. europaea fur *homologs

In order to determine which *fur *homolog of *N. europaea *encodes the Fe-sensing Fur protein, pFur616, pFur730 and pFur1722 plasmids (Table 1) were used to transform the *E. coli fur *mutant H1780 [40]. *E. coli *H1780 strain was engineered to be *fur *deficient and to include the Fur-regulated gene *fiu *fused to a promoterless *lacZ *gene. This reporter gene, *fiu-lacZ*, cannot be repressed in this strain due to the *fur *mutation, and therefore the gene encoding the enzyme β-galactosidase is constitutively expressed and the strain always shows Lac^+ ^phenotype [40]. The pFur616-kanC (Table 1) plasmid carrying kanamycin resistance cassette (Km^r^) insertion in the C-terminal region of NE0616 gene was used to transform H1780 as a negative control.

**Table 1:** Bacterial strains, plasmids and primers used in this study

Strains or plasmid	Description	Reference
***E. coli***		
DH5⟨	F2ø80d*lacZ*⊗M15 *endA1 recA1 gyrA96 thi-1 hsdR17(r_K_^- ^m_K_^+^) supE44 relA1 deoR Δ*(*lacZYA-argF*)U169	[56]
H1717	*aroB fhuF*::λp*lac*Mu	[40]
H1717 (pFur616)	*E. coli *H1717 carrying pFur616	This study
H1717 (pFur616-kanP)	*E. coli *H1717 carrying pFur616-kanP	This study
H1717 (pFur616-kanC)	*E. coli *H1717 carrying pFur616-kanC	This study
H1780	araD139∆^a^argF-lacU169rpsL150 relA1 flbB5301deoC1 ptsF25 rbsR fiu::lacZ fusion lacking Fur	[40]
H1780 (pFur616)	*E. coli *H1780 carrying pFur616	This study
H1780 (pFur616-kanP)	*E. coli *H1780 carrying pFur616-kanP	This study
H1780 (pFur616-kanC)	*E. coli *H1780 carrying pFur616-kanC	This study
H1780 (pFur730)	*E. coli *H1780 carrying pFur730	This study
H1780 (pFur1722)	*E. coli *H1780 carrying pFur1722	This study
***N. europaea***		
ATCC 19178	Wild type	ATCC
*fur:kanP*	Insertion of *kan *cassette in the *furbox *upstream of NE0616 gene	This study
**Plasmids**		
pGEM-T Easy	Vector for cloning PCR products; Amp^r^	Promega
pFur616	pGEM-T Easy vector containing NE0616 u&d* region	This study
pFur616-kanP	In vitro transposon mutagenesis of pFur616 with EZ-Tn5 <KAN-2> with k*an *cassette insertion in *fur box *located in promoter region of NE0616	This study
pFur616-kanC	In vitro transposon mutagenesis of pFur616 with EZ-Tn5 <KAN-2> with k*an *cassette insertion in C-terminal region of NE0616	This study
pFur730	pGEM-T Easy vector containing NE0730 u&d region	This study
pFur1722	pGEM-T Easy vector containing NE1722 u&d region	This study
**Primers used for cloning, mutagenesis and mutant confirmation**		
NE0616u&d-1	5'-ATCCTGGAAGAAAACGGTCA-3'	This study
NE0616u&d-2	5'-TGCAGGTTTCAAACGAAAAA-3'	This study
NE0730u&d-1	5'-TTTCAGACGTTGCTGACAAAA-3'	This study
NE0730u&d-2	5'-TCATTTTGGCTGTTCATTTCA-3'	This study
NE1722u&d-1	5'-TATGGCTTACGGAAAACGGTA-3'	This study
NE1722u&d-2	5'-ACAAAAACAGACACGGAGGAA-3'	This study

*- u&d denotes upstream & downstream region.

**Figure 3:**
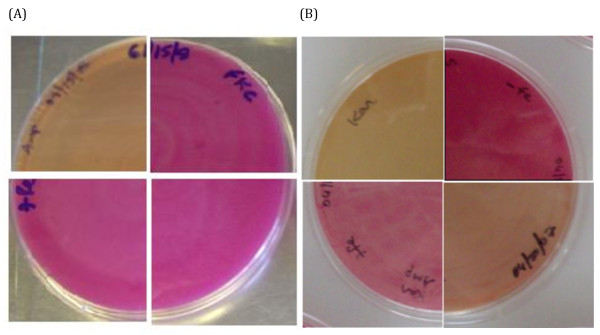
**Fur Titration Assays (FURTA)**. (A) Complementation of an *E. coli fur *mutant H1780 by *N. europaea *Fur homologs.*E. coli *H1780 (pFur616)-upper left quadrant; H1780 (pFur616-kanC)-upper right quadrant; H1780 (pFur730)-lower left quadrant; H1780 (pFur1722)-lower right quadrant plated on McConkey medium with 30 μM Fe supplement and grown at 37°C for 24 hrs. (B) *E. coli *H1717 plated on McConkey medium with 30 μM Fe supplement-upper left quadrant, no Fe supplement-upper right quadrant; H1717 (pFur616)-lower left quadrant; H1717 (pFur616-kanP)-lower right quadrant plated on McConkey medium with 30 μM Fe supplement and grown at 37°C for 24 hrs.

All strains evaluated for Lac phenotype were grown on McConkey Lactose plates with 30 μM iron supplement, since iron is required to ensure that Fur is functional as a repressor [6]. In these studies, *E. coli *H1780, H1780 (pFur616), H1780 (pFur616-kanC), H1780 (pFur730) and H1780 (pFur1722) strains were compared. Lac^+ ^phenotype was observed for *E. coli *H1780 whether grown in the presence or absence of added Fe supplement as predicted since it is deficient in Fur protein (data not shown). Complementation of *E. coli *H1780 with pFur616 rescued the Fur defect of this strain and resulted in the repression of transcription of the *fiu-lacZ *reporter gene, as shown by the Lac^- ^phenotype (Figure 3A; upper left quadrant). When pFur616-kanC plasmid containing the disrupted NE0616 gene, was transformed into the *E. coli *H1780 mutant, Lac^+ ^phenotype was maintained (Figure 3A; upper right quadrant). When pFur730 and pFur1722 plasmids containing the *N. europaea fur *homologs NE0730 and NE1722 were transformed separately into *E. coli *H1780 strain, Lac^+ ^phenotype was observed (Figure 3A; lower left and right quadrants). These results clearly demonstrate that the *N. europaea *NE0616 *fur *homolog is expressed in *E. coli *in a functional form and is capable of regulating the Fur-dependent *fiu *promoter in H1780. The other *N. europaea fur *homologs (NE0730 and NE1722) were not capable of regulating the *fiu *promoter in H1780. NE0616 is here after referred to as *N. europaea fur*.

### The *N. europaea fur *promoter is repressed by Fur

Several studies have employed *E. coli *H1717 strain to allow the detection of iron-regulated promoters in bacteria such as *E. coli *and *Salmonella typhimurium *[41,42]. *E. coli *H1717 strain has a chromosomal iron-regulated *fhuF *promoter fused to *lacZ*. This fusion is exceptionally sensitive to small changes in iron concentration because of the weak affinity of the *fhuF *promoter for the Fur-Fe^2+ ^repression complex. Introduction of a multi-copy plasmid carrying Fur-binding sites into the test strain depletes the intracellular Fur pool. This gives rise to the dissociation of the repressor from the fusion promoter, thereby allowing expression of enzyme β-galactosidase. We have screened plasmids pFur616 carrying intact Fur box and pFur616-kanP carrying disrupted Fur box using *E. coli *H1717 strain to determine NE0616 Fur box functionality. The pFur616-kanC plasmid (Table 1) carrying Km^r ^insertion in the C-terminal region of NE0616 gene was also used to transform *E. coli *H1717 as a positive control.

In these studies, *E. coli *H1717 in the presence and absence of Fe supplement, H1717 (pFur616), H1717 (pFur616-kanP) and H1717 (pFur616-kanC) strains were compared. Lac^- ^phenotype was observed for *E. coli *H1717 when grown in the presence of 30 μM Fe supplement, since it does not carry any multi-copy plasmid with a functional Fur box on it (Figure 3B upper left quadrant). Lac^+ ^phenotype was observed when H1717 was grown with no added Fe supplement, since there is not enough Fe to suppress *fhuF-lacZ *fusion (Figure 3B; upper right quadrant). When pFur616 carrying putative Fur box was transformed into *E. coli *H1717 and the resulting strain was grown in presence of 30 μM Fe supplement, it resulted in derepression of the *fhuF-lacZ *reporter gene, as shown by the Lac^+ ^phenotype (Figure 3B; lower left quadrant). This result indicates that the predicted Fur box is functional and must have titrated the intracellular Fur-Fe pool. When a pFur616-kanP plasmid containing the disrupted NE0616 Fur box, was transformed into the *E. coli *H1717 strain, Lac^- ^phenotype was restored (Figure 3B; lower right quadrant) indicating that the Km^r ^insertion led to disruption of Fur box functionality. When a pFur616-kanC plasmid containing Km^r ^insertion in the C-terminal region of NE0616 gene was transformed into *E. coli *H1717 strain, Lac^+ ^phenotype was observed (data not shown) indicating that Km^r ^in C-terminal region of NE0616 did not affect its Fur box functionality. These results demonstrate that the promoter of *N. europaea *NE0616 *fur *homolog carries a Fur box and it is functional as recognized by *E. coli *Fur protein.

### Isolation of the *N*. *europaea fur:kanP *mutant strain

**Figure 4:**
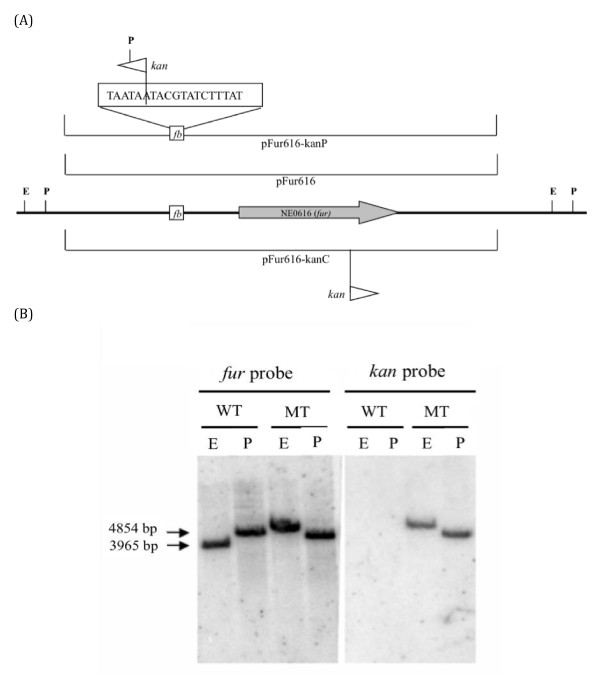
***In vitro *transposon mutagenesis scheme and mutant confirmation**. (A) The physical structure of a 5,810-bp fragment of the *N. europaea *chromosome is shown in the center (heavy black line), with positions of NE0616 (*fur*) gene shown as grey arrow, the fur box (*fb*) located in NE0616 promoter region shown as white rectangle. The regions covered by the plasmids pFur616, pFur616-kanP, pFur616-kanC whose DNA sequences were determined are shown as thin black lines with the names of the respective plasmids shown below each line. The position and relative orientation of each in vitro-constructed *Tn5-Kan2 *cassette insertion mutation are indicated by a flag on the lines. The restriction endonuclease sites P (*Pst*1) and E (*Eco*R1) used for Southern blot confirmation are indicated. (B) Verification of mutagenesis of *fur:kanP *in *N. europaea *by Southern hybridization. Genomic DNA from the wild type (WT), *fur:kanP *mutant (MT) were digested with E (*Eco*RI) and P (*Pst*1), and probed with (left) *fur *ORF sequence and (right) *kan *sequence.

To address the physiological role *fur *plays in *N. europaea*, we attempted to generate an *N. europaea fur *null mutant but were unsuccessful. However, we were successful in isolating an *N. europaea fur:kanP *mutant strain with Km^r ^inserted in the Fur box located in the promoter region of NE0616 gene (Figure 4A). The pFur616*-*kanP plasmid was electroporated into *N. europaea *wild-type cells. The *fur:kanP *mutant was obtained through homologous recombination and confirmed by PCR (data not shown) and Southern hybridization (Figure 4B). The *fur *probe detected a 3.96 Kb *Eco*R1 fragment and a 4.85 Kb *Pst*1 fragment in wild type and a ~ 5 Kb *Eco*R1 fragment and a ~ 4.3 Kb *Pst*1 fragment (calculated size based on the DNA sequences) in *fur:kanP *mutant strain. The kanamycin-cassette probe detected the same ~ 5 Kb *Eco*R1 fragment and the ~ 4.3 Kb *Pst*1 fragment in *fur:kanP *mutant but not in the wild type. These results confirm that a single copy of Km^r ^was correctly inserted in the Fur box located in the promoter region of NE0616 gene of the *N. europaea *genome (Figure 4A). A *fur *transcript was not detected in the *fur:kanP *mutant by either RT-PCR or qRT-PCR analysis (up to 28 cycles) indicating the inactivation of *fur *gene due to Km^r ^insertion in its promoter region. Transcripts of ammonia monooxygenase C (*amoC*) component used as positive control both for the efficiency of the RT-PCR procedure and for RNA and cDNA recovery showed no significant difference in expression in wild type and the *fur:kanP *mutant (data not shown).

### Effect of *fur:kanP *mutation on growth of *N. europaea*

**Figure 5:**
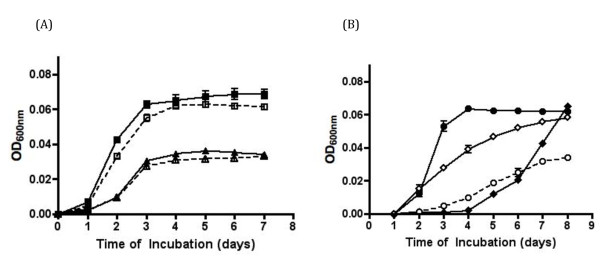
**Growth curves of the *N. europaea *wild type (solid lines, filled symbols) and *fur:kanP *mutant (dotted lines, open symbols) as measured by OD**. (A) Fe-replete (squares) and Fe-limited (triangles) medium. (B) 500 μM Fe medium (circles) and in Fe-limited medium with 10 μM ferrioxamine (diamonds). Data shown are means of triplicates, with variation less than 10%. The experiment was repeated several times and produced similar results. Error bars represent the standard error of the mean.

Growth of the *N. europaea fur:kanP *strain was compared to that of the wild-type strain in both Fe-replete (10 μM Fe) and Fe-limited (0.2 μM Fe) media. Surprisingly, there was no significant difference in growth of *fur:kanP *in both Fe-replete and Fe-limited media compared to the wild-type strain (Figure 5A). The *fur:kanP *mutant did not exhibit a growth advantage over the wild type when iron was limiting or show increased sensitivity to iron-induced redox stress when grown in the presence of Fe (up to 250 μM Fe; data not shown). However, growth of *fur:kanP *mutant was affected when grown in medium containing 500 μM Fe (Figure 5B). The mutant was unable to grow in media containing more than 500 μM Fe (data not shown). Growth of wild type was inhibited only when concentrations of Fe exceeded 1 mM [14].

*N. europaea *can use the siderophore ferrioxamine for its iron uptake after a 3 to 4 day lag period suggesting that the ferrioxamine uptake system in *N. europaea *requires induction [13,14]. When *N. europaea fur:kanP *mutant was grown in Fe-limiting media containing ferrioxamine, there was no lag phase (Figure 5B) indicating that the ferrioxamine uptake system was already induced in the *fur:kanP *mutant.

### Effect of *fur:kanP *mutation on induction of Fe-regulated outer membrane proteins in *N. europaea*

**Figure 6:**
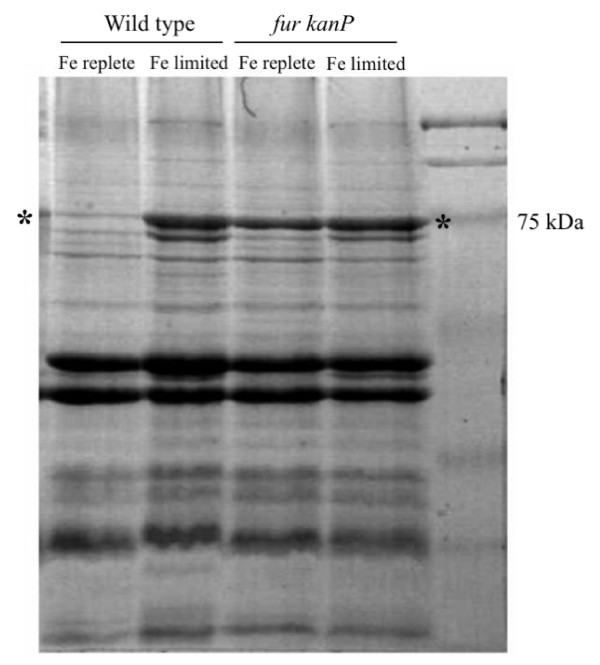
**SDS-PAGE Analysis of total membrane proteins**. *N. europaea *wild type and *fur:kanP *mutant in Fe-replete (10 μM) (lanes 1, 3) and Fe-limited (0.2 μM) media (lanes 2, 4). Over-expression of proteins with molecular weights similar to outer membrane Fe-siderophore receptors indicated by * was observed in *fur:kanP *mutant in both Fe-replete and Fe-limited media.

Previous studies have shown that *N. europaea *grown in Fe-limited medium stimulated expression of several Fe-regulated outer membrane proteins (TonB-dependent receptors) with molecular masses of ~ 80 kDa [13,14]. To determine whether the expression of these proteins was regulated by *fur*, the *N. europaea *wild type and the *fur:*kanP mutant strains were cultured in Fe-replete and Fe-limited media and their total outer membrane proteins were isolated. SDS-PAGE analysis of the outer membrane protein profiles demonstrated that *fur:kanP *mutant shared a major protein band (Figure 6) with wild type cells grown in Fe-limited media irrespective of the concentration of iron in the medium. This band contained several TonB-dependent OM Fe^3+^-siderophore receptors [13,14]. This result is consistent with the model in which the TonB-dependent receptors with putative roles in iron uptake are regulated by *fur *[6].

### Effect of *fur:kanP *mutation on Fe and heme *c *contents of *N. europaea*

Fur deficient mutants generally express iron transport systems constitutively (with respect to iron), and have increased free cellular iron levels (although total cellular iron levels are actually reduced, due to low levels of iron-storage and iron-containing proteins) [43,44]. To determine the effect of *fur:kanP *mutation on iron contents of *N. europaea*, wild type and *fur:kanP *mutant cells were cultured in Fe-replete and Fe-limited media and their total cellular iron contents were measured by ICP-OES analysis. *N. europaea *Fe-limited cells showed significantly (P-value <0.0001) lower total cellular iron contents compared to Fe-replete cells irrespective of the *fur *mutation as observed previously (Table 2) [14]. The *fur:kanP *mutant had 1.5-fold significantly (P-value <0.001) more total cellular iron than the wild-type cells when grown in Fe-replete media (Table 2). The total iron contents of wild type and the *fur:kanP *mutant did not show significant (P-value = 0.47) variation when grown in Fe-limited medium (Table 2). The *fur:kanP *mutation also influenced both the amount of soluble cytochromes produced and the proportion of iron distributed to cytochromes (Table 2). These data suggest that in *N. europaea*, Fur regulates the concentration of intracellular iron through modulation of iron acquisition and iron consumption, and that, in the absence of Fur, *N. europaea *is unable to regulate its iron acquisition.

**Table 2:** Physiological characteristics of *N. europae a *wild type and *fur:kanP *mutant grown under Fe-replete (10 μM) and Fe-limited (0.2 μM) conditions*

Physiological Characteristic	Wild type		*fur:kanP *mutant	
	
	Fe-replete	Fe-limited	Fe-replete	Fe-limited
				
**Heme *c *content in cell's** **soluble fraction**				
				
Heme *c *(nmol/ml culture)	0.85 ± 0.02	0.38 ± 0.05	0.48 ± 0.02	0.21 ± 0.04
Heme *c *(nmol/mg protein)	7.77 ± 0.23	4.04 ± 0.53	5.67 ± 0.31	5.04 ± 0.91
				
**Whole Cell Fe content**				
				
Fe (nmol/ml culture)	1.36 ± 0.15	0.15 ± 0.01	2.04 ± 0.09	0.11 ± 0.01
Fe (nmol/mg protein)	90.4 ± 6.0	26.4 ± 2.0	136.2 ± 14.0	24.9 ± 3.0
Cellular Fe concentration (mM)	8.27 ± 0.94	1.99 ± 0.13	12.4 ± 0.6	1.98 ± 0.18
				
**Whole cell enzyme-catalyzed activity**				
				
NH_4_^+^-dependent O_2 _consumption (nmol/(min × OD_600 nm_)	94.5 ± 4.1	38.1 ± 6.0	88.2 ± 2.5	21.7 ± 0.6
NH_4_^+^-dependent O_2 _consumption (nmol/(min × mg protein)	1500 ± 63	779 ± 17	1446 ± 40	680 ± 18
				
NH_2_OH-dependent O_2 _consumption (nmol/(min × OD_600 nm_)	25.9 ± 0.2	10.9 ± 2.4	25.7 ± 4.8	4.6 ± 0.2
NH_2_OH-dependent O_2 _consumption (nmol/(min × mg protein)	412 ± 3.0	222 ± 5.0	421 ± 2.0	146 ± 6.0

*Data are means of triplicates, with variation less than 10%. The experiment was repeated several times and produced similar results. Data are means ± S.D.

### Effect of *fur:kanP *mutation on NH_4_^+^- and NH_2_OH-dependent O_2 _uptake activities of *N. europaea*

As indicators of the overall cell activity, NH_4+_- and NH_2_OH-dependent O_2 _uptake rates in wild type and *fur:kanP *mutant cells grown in Fe-replete and Fe-limited media were measured. *N. europaea *Fe-limited cells showed significantly (P-value <0.0001) lower activities compared to Fe-replete cells irrespective of the *fur *mutation as observed previously (Table 2) [14]. The activities of wild type and *fur:kanP *mutant strains did not show significant (P-value ≤ 0.4) variation when grown in Fe-replete media (Table 2). The NH_4+_-dependent O_2 _uptake activities, which require both ammonia monooxygenase and hydroxylamine oxidoreductase activity, when measured at per mg basis were not affected; however the NH_2_OH-dependent O_2 _uptake activity, which requires hydroxylamine oxidoreductase, but not ammonia monooxygenase activity, was significantly (P-value <0.0001) two-fold lower in *fur:kanP *Fe-limited cells compared to wild type Fe-limited cells (Table 2). This result is consistent with our observation of lower heme contents in *fur:kanP *mutant than wild type. Hydroxylamine oxidoreductse contains 24 hemes per enzyme [45] and the lower NH_2_OH-dependent O_2 _uptake activity in Fe-limited cells of *fur:kanP *mutant than wild type might be due to the low availability of heme under Fe-limited conditions. This data also suggests that the *fur:kanP *mutation led to an improper balance of iron allocation in *N. europaea*.

## Discussion

We provide several lines of evidence that the Fur homolog encoded by *N. europaea *gene NE0616 is the Fe-sensing Fur protein. First, we have shown that NE0616 shares all eight of the metal binding amino acid residues of *P. aeruginosa *Fur (Figure 1) [19] and that the Fur homolog encoded by NE0616 is clustered with Fe-sensing Fur proteins from other bacteria (Figure 2). An *E. coli *Fur titration assay (FURTA) system for Fur analysis was utilized as a second method to confirm that the cloned NE0616 *fur *encodes a functional protein. The H1780 (pFur616) strain carrying NE0616 *fur *homolog on a plasmid was evaluated for its ability to utilize lactose as described by Hantke et al., [40]. Utilization of lactose by H1780 (pFur616) strain was detected by color change of colonies from white to red on McConkey lactose plates indicating the formation of lactic acid. Lactose utilization was not detected when H1780 strain carrying plasmids pFur616-kanC, pFur730, pFur1722 were plated on McConkey lactose plates (Figure 3A).

One of the major limitations in our research on the role of Fur has been the inability to make a *fur *null mutant. Null mutations have been successfully isolated for *E. coli *[46,47], *V. cholerae *[48], *Shigella flexneri *[49], *Neisseria meningitidis *[34]. Unsuccessful attempts to isolate insertional null mutants were reported for *P. aeruginosa *[50], *Pseudomonas putida *[51], and *N. gonorrhoeae *[52]. To date, multiple attempts to generate a *N. europaea fur *mutant have been unsuccessful. Loss of the *fur *gene may be a lethal mutation in *N. europaea*, as occurs in some other gram-negative bacteria [50]. However, we were successful in generating an *N. europaea fur *promoter knockout mutant (*fur:kanP*) (Figure 4A). Southern analysis with probes internal to *fur *or the Km^r ^corroborated insertion of Km^r ^in the promoter region of the *fur *gene (Figure 4B) and hence *fur:kanP *mutant strain was selected for further analysis. Although we were unable to detect the NE0616 transcript in *fur:kanP *mutant strain by RT-PCR or qRT-PCR, it is possible that there is some leaky transcription of *fur *in our mutant strain, since it is a promoter knockout mutant. This could be the reason why we were able to generate a promoter knockout mutant but not a *fur *null mutant.

The effects of *fur:kanP *mutation on *N. europaea *were broad. Inactivation of the *fur *gene (resulting in deregulation of iron metabolism) increases sensitivity to redox stress when grown under iron-rich conditions in some bacteria such as *E. coli *[53]. The *N. europaea*, wild-type and the *fur:kanP *mutant strain showed similar growth patterns when grown in Fe-replete (10 μM Fe) and Fe-limited (0.2 μM Fe) media (Figure 5A). However, the *fur:kanP *mutant did not grow well when cultured in media containing 500 μM iron (Figure 5B). The *fur:kanP *mutant was unable to grow beyond 500 μM Fe concentrations while the wild-type strain was able to withstand iron concentrations up to 1 mM (data not shown). These results indicate that *N. europaea *Fur plays a role in regulating uptake of iron when present in excess and also probably helps to overcome oxidative stress.

Increased intracellular free iron is likely to result from deregulated iron uptake by the *fur *mutant [43]. The *N. europaea fur:kanP *mutant strain grown to mid exponential phase in Fe-replete media (10 μM Fe) contained 1.5-fold higher total cellular iron than that of the wild-type strain as measured by ICP-OES (Table 2). Our measurements of total acid-soluble non-heme iron cannot distinguish between free iron and iron bound to proteins. Hence we measured the heme contents of wild type and *fur:kanP *mutant strains and observed that the *fur:kanP *mutant had 1.4-fold lower heme contents compared to wild type (Table 2). In addition, the activity of iron-rich hydroxylamine oxidoreductase enzyme was lower in *fur:kanP *mutant strain (Table 2). These results indicated that the balance between acquiring enough iron and allocating it to various Fe-dependent proteins is lost in *N. europaea fur:kanP *mutant.

*N. europaea *protein profiles showed over expression of several outer membrane proteins upon Fe-limitation [13,14]. We have observed similar over expression of outer membrane proteins in *N. europaea fur:kanP *mutant (Figure 6 band indicated by *) irrespective of iron availability. These data are consistent with previous studies describing *fur *mutations in other bacterial species [54,55].

## Conclusions

In summary, we have identified and characterized through insertional inactivation one of the three *N. europaea *Fur homologs. The *N. europaea *Fur protein encoded by gene NE0616 has extensive homology to the *E. coli *Fur protein and was able to complement an *E. coli fur *mutant. The *N. europaea fur:kanP *mutant is unable to regulate its intracellular iron and heme concentrations and appears to induce its iron acquisition systems constitutively. Additional studies are required to fully delineate the role of this *N. europaea fur *homolog.

## Methods

### Bacterial cultures and siderophore feeding experiments

*N. europaea *(ATCC 19178) was cultured as described with minor modifications [22,23]. The standard (Fe-replete) medium contained 10 μM Fe^3+ ^(FeCl_3_) complexed with EDTA to prevent Fe precipitation. Fe-limited medium was made from reagent-grade chemicals, without addition of any Fe salt, and contained 0.2 μM Fe [14]. All media, buffers and other reagents were made in double-deionized water. All glassware was soaked in 1% HNO_3 _overnight, and then rinsed thoroughly with double-deionized water. Fe-free Desferal (deferoxamine/DFX mesylate) was purchased from Sigma (St. Louis, MO). Desferal was dissolved in double deionized water, filter sterilized, and added to Fe-limited medium in the siderophore feeding experiments. In this study 10 μM Desferal was used to ensure the complete chelation of Fe (0.2 μM) in the Fe-limited medium. *N. europaea *cultures were grown at 30°C on a rotary shaker, and mid-exponential-phase cells were collected by centrifugation and thorough washes for the analyses. *E. coli *DH5α, *E. coli *H1780 strain lacking *fur *gene, and *E. coli *H1717 strain were cultured on Luria-Bertani (LB) agar plates or in liquid LB medium in the presence of the appropriate antibiotic (ampicillin [100 μg ml^-1^] and/or kanamycin [20 μg ml^-1^]) under the conditions described above.

### DNA preparation, PCR, cloning, mutagenesis and mutant isolation

General DNA preparation, restriction digestions and agarose gel electrophoresis were done as described by [24]. The three *N. europaea fur *homologs (Figure 1) were amplified by PCR using Taq DNA polymerase (Promega, Madison, WI) on an iCycler Thermal Cycler (Bio-Rad, Hercules, CA), as described by the manufacturers (see Table 1 for primers). The resulting DNA fragments were cloned into the pGEM-T Easy vector (Promega), sequenced to confirm that no mutations have been introduced and named pFur616, pFur730 and pFur1722 respectively. *E. coli *DH5α was used for plasmid amplification. For insertion of kanamycin resistance cassette (Km^r^) into plasmid pFur616, the EZ::TN <KAN2> kit from Epicentre (Madison, WI) was used to insert a transposon conferring Km^r ^into the promoter region (pFur-kanP) and C-terminal region (pFur-kanC) of *fur *following the directions of the manufacturer. The insertion of the Km^r ^gene was localized by nucleotide sequence determination at 117 nt upstream of the ATG start codon of *fur *(pFur-kanP) and 312 nt downstream of the ATG start codon of *fur *(pFur-kanC) in plasmid pFur616. The pFur616-kanP plasmid construct with the Km^r ^insertion was introduced back into the *N. europaea *wild type cells by electroporation on the ElectroPorator (Invitrogen, Carlsbad, CA) at 1300 V, with a capacitance at 50 μF, and a load resistance at 500 Ω. Successful transformants were selected in liquid medium using kanamcyin sulfate (20 μg ml^-1^). Aliquots from these cultures were streaked onto Nylon disk membranes, which were placed on semisolid plates, to isolate clonal mutant strains, as described [25]. The mutant was verified by Southern analysis (Figure 4B, and Results). Southern blotting, labeling of DNA probes, hybridization and imaging were done as described previously [26]. Attempts to generate *fur *null mutant by using pFur-kanC construct were unsuccessful.

### Fur Titration Assays (FURTA)

Plasmids (listed in Table 1) were introduced into *E. coli *H1717 and H1780 (*fur *inactivated) strains and *lacZ *expression was assessed by visualization of a change in colony color from white to red on MacConkey lactose plates (Difco) supplemented with 30 μM ferrous ammonium sulfate. Plates were examined after 24 h of growth at 37°C. The assays were performed in triplicate for each sample.

### Determination of Fe and heme *c *contents and O_2_-uptake activities

Total Fe contents in thoroughly washed *N. europaea *cells were determined by the ferrozine assay following HNO_3 _(5%) digestion of cells at 100°C [27]. Measurements of Fe concentrations below 10 μM were made using a Teledyne Leeman Prodigy ICP-OES (Hudson, NH) at the W.M. Keck Collaboratory for Plasma Spectrometry, Oregon State University. Preparations of a cell-soluble fraction, and determination of heme contents following extraction with pyridine, were done as described [14,28]. Whole cell NH_3_-dependent and hydroxylamine dependent O_2 _uptake activities were measured as described [14,29]. The significance (P-values) for the physiological changes of the strains due to the treatments (Table 2) was assessed using Student's t-test. The P-values below 0.01 were considered statistically significant.

### Cell fractionation, protein quantification and SDS-PAGE analyses

Total cell membranes were prepared as previously described [14]. Briefly, cells were broken by ultrasonication, the sonicated material was centrifuged at 1500 g for 1 min to pellet unlysed cells, and the top phase (cell lysate) was transferred to ultracentrifuge tubes. Crude total membranes were collected by ultracentrifugation of the cell lysates, and washed thoroughly by homogenization in Tris buffer (0.1 M, pH 7.8) containing 1 M KCl. Total membranes were collected again by ultracentrifugation, and resuspended in Tris buffer (50 mM, pH 7.8). Protein contents in whole cells and cell fractions were estimated by using the Micro BCA Protein Assay kit (Pierce), and BSA was used as a protein standard. The peptide composition of cell membranes was analyzed using SDS-PAGE [with 12% (w/v) acrylamide in the resolving gels], as described [14,30].

### Phylogenetic tree construction

ClustalW was used for sequence alignment applying default parameters (altered gap penalties were not applied) [31]. Gaps in the alignment were not omitted. The phylogenetic tree was built by Phyml 3.0 with the distance matrix generated by ClustalW and was represented with the program TreeDyn 198.3 available at http://www.phylogeny.fr/[32]. The reliability of each node was established by bootstrap methods.

### Hidden Markov Model-based Fur binding site prediction

A group of experimentally validated Fur boxes from *E. coli*, *S. typhimurium*, *P. aeruginosa *and *S. aureus *used by Quatrini et al., [33] along with 3 experimentally confirmed *N. europaea *Fur boxes were used to build HMM profiles and to search for fur binding sites in the promoter regions (600 nucleotides upstream of the proposed initiation of translation) of the potential target genes. Alignment of these promoters with the ClustalW multiple-sequence alignment program yielded a putative *Nitrosomonas *Fur box consensus sequence that has 80% homology with the *E. coli *Fur box consensus binding sequence.

*N. europaea *sequence data was obtained from DOE Joint Genome Institute (JGI) website http://genome.ornl.gov/microbial/neur/. Sequence similarity searches of the available nucleotide and protein databases were performed with the BLAST program, available at the National Center for Biotechnology Information website http://www.ncbi.nlm.nih.gov/blast/.

## Authors' contributions

NV, LS, PB and DA conceived the study and participated in its design and coordination. NV collected and analyzed the data and wrote the manuscript. LS, PB and DA assisted in the drafting and provided substantial editorial advice and a critical revision of the manuscript. All authors have read and approved the manuscript.

## References

[B1] Hantke K Cloning of the repressor protein gene of iron-regulated systems in *Escherichia coli *K12 Mol Gen Genet 1984 197 2 337 341 10.1007/BF00330982 6097798

[B2] Ernst FD Bereswill S Waidner B Stoof J Mader U Kusters JG Kuipers EJ Kist M van Vliet AH Homuth G Transcriptional profiling of *Helicobacter pylori *Fur- and iron-regulated gene expression Microbiology 2005 151 Pt 2 533 546 10.1099/mic.0.27404-0 15699202

[B3] Holmes K Mulholland F Pearson BM Pin C McNicholl-Kennedy J Ketley JM Wells JM *Campylobacter jejuni *gene expression in response to iron limitation and the role of Fur Microbiology 2005 151 Pt 1 243 257 10.1099/mic.0.27412-0 15632442

[B4] McHugh JP Rodriguez-Quinones F Abdul-Tehrani H Svistunenko DA Poole RK Cooper CE Andrews SC Global iron-dependent gene regulation in *Escherichia coli*. A new mechanism for iron homeostasis J Biol Chem 2003 278 32 29478 29486 10.1074/jbc.M303381200 12746439

[B5] Mey AR Wyckoff EE Kanukurthy V Fisher CR Payne SM Iron and *fur *regulation in *Vibrio cholerae *and the role of *fur *in virulence Infect Immun 2005 73 12 8167 8178 10.1128/IAI.73.12.8167-8178.2005 16299312 PMC1307094

[B6] Escolar L Perez-Martin J de Lorenzo V Opening the iron box: transcriptional metalloregulation by the Fur protein J Bacteriol 1999 181 20 6223 6229 10515908 10.1128/jb.181.20.6223-6229.1999 PMC103753

[B7] Lee JW Helmann JD Functional specialization within the Fur family of metalloregulators Biometals 2007 20 3-4 485 499 10.1007/s10534-006-9070-7 17216355

[B8] Crosa JH Genetics and molecular biology of siderophore-mediated iron transport in bacteria Microbiol Rev 1989 53 4 517 530 2531838 10.1128/mr.53.4.517-530.1989 PMC372751

[B9] Chain P Lamerdin J Larimer F Regala W Lao V Land M Hauser L Hooper A Klotz M Norton J Complete genome sequence of the ammonia-oxidizing bacterium and obligate chemolithoautotroph *Nitrosomonas europaea* J Bacteriol 2003 185 9 2759 2773 10.1128/JB.185.9.2759-2773.2003 12700255 PMC154410

[B10] Whittaker M Bergmann D Arciero D Hooper AB Electron transfer during the oxidation of ammonia by the chemolithotrophic bacterium *Nitrosomonas europaea* Biochim Biophys Acta 2000 1459 2-3 346 355 10.1016/S0005-2728(00)00171-7 11004450

[B11] Upadhyay AK Petasis DT Arciero DM Hooper AB Hendrich MP Spectroscopic characterization and assignment of reduction potentials in the tetraheme cytochrome C554 from *Nitrosomonas europaea* J Am Chem Soc 2003 125 7 1738 1747 10.1021/ja020922x 12580599

[B12] Schwyn B Neilands JB Universal chemical assay for the detection and determination of siderophores Anal Biochem 1987 160 1 47 56 10.1016/0003-2697(87)90612-9 2952030

[B13] Wei X Sayavedra-Soto LA Arp DJ Characterization of the ferrioxamine uptake system of *Nitrosomonas europaea* Microbiology 2007 153 Pt 12 3963 3972 10.1099/mic.0.2007/010603-0 18048911

[B14] Wei X Vajrala N Hauser L Sayavedra-Soto LA Arp DJ Iron nutrition and physiological responses to iron stress in *Nitrosomonas europaea* Arch Microbiol 2006 186 2 107 118 10.1007/s00203-006-0126-4 16802173

[B15] Patzer SI Hantke K The ZnuABC high-affinity zinc uptake system and its regulator Zur in *Escherichia coli* Mol Microbiol 1998 28 6 1199 1210 10.1046/j.1365-2958.1998.00883.x 9680209

[B16] Bsat N Herbig A Casillas-Martinez L Setlow P Helmann JD *Bacillus subtilis *contains multiple Fur homologues: identification of the iron uptake (Fur) and peroxide regulon (PerR) repressors Mol Microbiol 1998 29 1 189 198 10.1046/j.1365-2958.1998.00921.x 9701813

[B17] Hernandez JA Lopez-Gomollon S Bes MT Fillat MF Peleato ML Three *fur *homologues from *Anabaena sp. PCC7120*: exploring reciprocal protein-promoter recognition FEMS Microbiol Lett 2004 236 2 275 282 10.1111/j.1574-6968.2004.tb09658.x 15251208

[B18] Gaballa A Helmann JD Identification of a zinc-specific metalloregulatory protein, Zur, controlling zinc transport operons in *Bacillus subtilis* J Bacteriol 1998 180 22 5815 5821 9811636 10.1128/jb.180.22.5815-5821.1998 PMC107652

[B19] Pohl E Haller JC Mijovilovich A Meyer-Klaucke W Garman E Vasil ML Architecture of a protein central to iron homeostasis: crystal structure and spectroscopic analysis of the ferric uptake regulator Mol Microbiol 2003 47 4 903 915 10.1046/j.1365-2958.2003.03337.x 12581348

[B20] Patzer SI Hantke K The zinc-responsive regulator Zur and its control of the *znu *gene cluster encoding the ZnuABC zinc uptake system in *Escherichia coli* J Biol Chem 2000 275 32 24321 24332 10.1074/jbc.M001775200 10816566

[B21] Hall HK Foster JW The role of fur in the acid tolerance response of *Salmonella typhimurium *is physiologically and genetically separable from its role in iron acquisition J Bacteriol 1996 178 19 5683 5691 8824613 10.1128/jb.178.19.5683-5691.1996 PMC178407

[B22] Ensign SA Hyman MR Arp DJ In vitro activation of ammonia monooxygenase from *Nitrosomonas europaea *by copper J Bacteriol 1993 175 7 1971 1980 8458839 10.1128/jb.175.7.1971-1980.1993 PMC204278

[B23] Stein LY Arp DJ Loss of ammonia monooxygenase activity in *Nitrosomonas europaea *upon exposure to nitrite Appl Environ Microbiol 1998 64 10 4098 4102 9758853 10.1128/aem.64.10.4098-4102.1998 PMC106612

[B24] Sambrook J Fritsch EF Maniatis T Molecular Cloning: a Laboratory Manual 1989 2 Cold Spring Harbor, NY: Cold Spring Harbor Laboratory

[B25] Hommes NG Sayavedra-Soto LA Arp DJ Mutagenesis of hydroxylamine oxidoreductase in *Nitrosomonas europaea *by transformation and recombination J Bacteriol 1996 178 13 3710 3714 8682770 10.1128/jb.178.13.3710-3714.1996 PMC232626

[B26] Wei X Sayavedra-Soto LA Arp DJ The transcription of the *cbb *operon in *Nitrosomonas europaea* Microbiology 2004 150 Pt 6 1869 1879 10.1099/mic.0.26785-0 15184573

[B27] Carter P Spectrophotometric determination of serum iron at the submicrogram level with a new reagent (ferrozine) Anal Biochem 1971 40 2 450 458 10.1016/0003-2697(71)90405-2 5551554

[B28] Berry EA Trumpower BL Simultaneous determination of hemes a, b, and c from pyridine hemochrome spectra Anal Biochem 1987 161 1 1 15 10.1016/0003-2697(87)90643-9 3578775

[B29] Shiemke AK Arp DJ Sayavedra-Soto LA Inhibition of membrane-bound methane monooxygenase and ammonia monooxygenase by diphenyliodonium: implications for electron transfer J Bacteriol 2004 186 4 928 937 10.1128/JB.186.4.928-937.2004 14761987 PMC344235

[B30] Hyman MR Arp DJ An electrophoretic study of the thermal- and reductant-dependent aggregation of the 27 kDa component of ammonia monooxygenase from *Nitrosomonas europaea* Electrophoresis 1993 14 7 619 627 10.1002/elps.1150140197 8375353

[B31] Thompson JD Higgins DG Gibson TJ CLUSTAL W: improving the sensitivity of progressive multiple sequence alignment through sequence weighting, position-specific gap penalties and weight matrix choice Nucleic Acids Res 1994 22 22 4673 4680 10.1093/nar/22.22.4673 7984417 PMC308517

[B32] Dereeper A Guignon V Blanc G Audic S Buffet S Chevenet F Dufayard JF Guindon S Lefort V Lescot M Phylogeny.fr: robust phylogenetic analysis for the non-specialist Nucleic Acids Res 2008 36 Web Server W465 469 10.1093/nar/gkn180 18424797 PMC2447785

[B33] Quatrini R Lefimil C Veloso FA Pedroso I Holmes DS Jedlicki E Bioinformatic prediction and experimental verification of Fur-regulated genes in the extreme acidophile *Acidithiobacillus ferrooxidans* Nucleic Acids Res 2007 35 7 2153 2166 10.1093/nar/gkm068 17355989 PMC1874648

[B34] Delany I Ieva R Alaimo C Rappuoli R Scarlato V The iron-responsive regulator *fur *is transcriptionally autoregulated and not essential in *Neisseria meningitidis* J Bacteriol 2003 185 20 6032 6041 10.1128/JB.185.20.6032-6041.2003 14526014 PMC225026

[B35] Delany I Spohn G Pacheco AB Ieva R Alaimo C Rappuoli R Scarlato V Autoregulation of *Helicobacter pylori *Fur revealed by functional analysis of the iron-binding site Mol Microbiol 2002 46 4 1107 1122 10.1046/j.1365-2958.2002.03227.x 12421315

[B36] Ochsner UA Vasil ML Gene repression by the ferric uptake regulator in *Pseudomonas aeruginosa*: cycle selection of iron-regulated genes Proc Natl Acad Sci USA 1996 93 9 4409 4414 10.1073/pnas.93.9.4409 8633080 PMC39551

[B37] Desai PJ Angerer A Genco CA Analysis of Fur binding to operator sequences within the *Neisseria gonorrhoeae **fbpA *promoter J Bacteriol 1996 178 16 5020 5023 8759870 10.1128/jb.178.16.5020-5023.1996 PMC178289

[B38] Watnick PI Butterton JR Calderwood SB The interaction of the *Vibrio cholerae *transcription factors, Fur and IrgB, with the overlapping promoters of two virulence genes, irgA and irgB Gene 1998 209 1-2 65 70 10.1016/S0378-1119(98)00018-3 9524224

[B39] Baichoo N Helmann JD Recognition of DNA by Fur: a reinterpretation of the Fur box consensus sequence J Bacteriol 2002 184 21 5826 5832 10.1128/JB.184.21.5826-5832.2002 12374814 PMC135393

[B40] Hantke K Selection procedure for deregulated iron transport mutants (fur) in *Escherichia coli *K 12: fur not only affects iron metabolism Mol Gen Genet 1987 210 1 135 139 10.1007/BF00337769 3323834

[B41] Stojiljkovic I Baumler AJ Hantke K Fur regulon in gram-negative bacteria. Identification and characterization of new iron-regulated *Escherichia coli *genes by a fur titration assay J Mol Biol 1994 236 2 531 545 10.1006/jmbi.1994.1163 8107138

[B42] Tsolis RM Baumler AJ Stojiljkovic I Heffron F Fur regulon of *Salmonella typhimurium*: identification of new iron-regulated genes J Bacteriol 1995 177 16 4628 4637 7642488 10.1128/jb.177.16.4628-4637.1995 PMC177226

[B43] Abdul-Tehrani H Hudson AJ Chang YS Timms AR Hawkins C Williams JM Harrison PM Guest JR Andrews SC Ferritin mutants of *Escherichia coli *are iron deficient and growth impaired, and *fur *mutants are iron deficient J Bacteriol 1999 181 5 1415 1428 10049371 10.1128/jb.181.5.1415-1428.1999 PMC93529

[B44] Keyer K Imlay JA Superoxide accelerates DNA damage by elevating free-iron levels Proc Natl Acad Sci USA 1996 93 24 13635 13640 10.1073/pnas.93.24.13635 8942986 PMC19375

[B45] Arciero DM Hooper AB Hydroxylamine oxidoreductase from *Nitrosomonas europaea *is a multimer of an octa-heme subunit J Biol Chem 1993 268 20 14645 14654 8325841

[B46] Bagg A Neilands JB Mapping of a mutation affecting regulation of iron uptake systems in *Escherichia coli K-12* J Bacteriol 1985 161 1 450 453 3918009 10.1128/jb.161.1.450-453.1985 PMC214895

[B47] Hantke K Regulation of ferric iron transport in *Escherichia coli *K12: isolation of a constitutive mutant Mol Gen Genet 1981 182 2 288 292 10.1007/BF00269672 7026976

[B48] Litwin CM Calderwood SB Analysis of the complexity of gene regulation by *fur *in *Vibrio cholerae* J Bacteriol 1994 176 1 240 248 8282702 10.1128/jb.176.1.240-248.1994 PMC205036

[B49] Schmitt MP Payne SM Genetics and regulation of enterobactin genes in *Shigella flexneri* J Bacteriol 1988 170 12 5579 5587 2973458 10.1128/jb.170.12.5579-5587.1988 PMC211654

[B50] Prince RW Cox CD Vasil ML Coordinate regulation of siderophore and exotoxin A production: molecular cloning and sequencing of the *Pseudomonas aeruginosa fur *gene J Bacteriol 1993 175 9 2589 2598 8478325 10.1128/jb.175.9.2589-2598.1993 PMC204560

[B51] Venturi V Ottevanger C Bracke M Weisbeek P Iron regulation of siderophore biosynthesis and transport in *Pseudomonas putida WCS358*: involvement of a transcriptional activator and of the Fur protein Mol Microbiol 1995 15 6 1081 1093 10.1111/j.1365-2958.1995.tb02283.x 7623664

[B52] Thomas CE Sparling PF Isolation and analysis of a *fur *mutant of *Neisseria gonorrhoeae* J Bacteriol 1996 178 14 4224 4232 8763952 10.1128/jb.178.14.4224-4232.1996 PMC178181

[B53] Andrews SC Robinson AK Rodriguez-Quinones F Bacterial iron homeostasis FEMS Microbiol Rev 2003 27 2-3 215 237 10.1016/S0168-6445(03)00055-X 12829269

[B54] Horsburgh MJ Ingham E Foster SJ In *Staphylococcus aureus*, *fur *is an interactive regulator with PerR, contributes to virulence, and Is necessary for oxidative stress resistance through positive regulation of catalase and iron homeostasis J Bacteriol 2001 183 2 468 475 10.1128/JB.183.2.468-475.2001 11133939 PMC94901

[B55] Staggs TM Fetherston JD Perry RD Pleiotropic effects of a *Yersinia pestis **fur *mutation J Bacteriol 1994 176 24 7614 7624 8002585 10.1128/jb.176.24.7614-7624.1994 PMC197218

[B56] Hanahan D Studies on transformation of *Escherichia coli *with plasmids J Mol Biol 1983 166 4 557 580 10.1016/S0022-2836(83)80284-8 6345791

